# Acute Liver Failure and Myelosuppression due to Parvovirus B19 Infection: A Case Report

**DOI:** 10.4021/gr2009.03.1278

**Published:** 2009-03-20

**Authors:** Jian Qi Lian, Ye Zhang, Li Liu, Xian Guang Bai, Zhi Jun Liang, Yan Yan Zhao, Ying Min Liang

**Affiliations:** a Center of Infectious Diseases, ^b^Department of Hemotology, Tangdu Hospital, The Fourth Military Medical University, Xi'an, Shaanxi province, China

**Keywords:** Parvovirus B19, Acute Liver Failure, Myelosuppression

## Abstract

Parvovirus B19 has been associated with different diseases, such as erythema infectiosum, arthropathy and transient aplastic crisis. However, parvovirus B19 infection presenting as hepatic dysfunction and myelosuppression is rarely reported in adult patients. Herein, we report an adult case of acute parvovirus B19 infection presented with acute liver failure and myelosuppression. After being treated with gamma globulin, the patient’s liver function and bone marrow test improved. We conclude that the parvovirus B19 infection should be considered as a possible cause of acute liver injury and bone marrow suppression. The antibody for B19 virus should be routinely tested in patients with liver dysfunction and/or myelosuppression of unclear etiology.

## Introduction

Parvovirus B19, a member of the family *Parvoviridae*, subfamily *Parvovirinae*, genus *Erythrovirus*, is a small, single-stranded DNA virus with approximately 5,000 nucleotides that codes for two major structural or capsid proteins, VP1 and VP2, and one nonstructural protein, NS1. B19 virus has been implicated in a wide spectrum of illnesses, which are transmitted via blood products or through aerosolized droplets and fomite contamination. B19 causes erythema infectiosum (Fifth disease), which has been suggested as one of the causes of acute non-A, non-E hepatitis in children infection [1]. Parvovirus B19 infection in adults often manifests as arthropathy, particularly in females [2]. B19 infection is also associated with transient aplastic crisis by destroying the erythroid precursor pool in patients with chronic hemolytic anemia, such as sickle cell disease or hereditary spherocytosis [3]. In immunocompromised patients, symptomatic B19 infection can persist for months or even years [4]. On the other hand, asymptomatic infection has been reported to occur in about one-quarter of immunocompetent adults [5]. Most infections occur during childhood, resulting in anti-B19 IgG prevalence of about 60% at the age of 12 years [6]. Uncommon manifestations of B19 infection include dermatological, hematological, vascular, and neurological presentations [7]. Liver involvements in B19 infection are uncommon, especially in adult patients [8]. Herein, we report an adult case of parvovirus B19 infection with acute liver failure and myelosuppression.

## Case Report

A 34-year-old female, without any positive medical history, reported malaise and fever of 23 days, and jaundice of 1 week. In addition to sporadic doses of paracetamol and penicillin, she had not received any other treatment before admission. Examination upon admission revealed slight hepatomegaly and splenomegaly. Maculopapules were found in cervical and thoracic areas. However, no anemia, palpable cervical lymph nodes or other remarkable physical signs were observed. Blood alanine aminotransferase (ALT) was 307 U/L (normal range, NR, 0-40 U/L); aspartate aminotransferase (AST), 444 U/L (NR, 0-30 U/L); gamma-glutamyl transferase (GGT), 97 U/L (NR, 0-50 U/L); alkaline phosphatase (ALP), 142 U/L (NR, 45-132U/L); total bilirubin (TBIL), 195.70 µmol/L (NR, 3.0-22.0 µmol/L); direct bilirubin (DB), 183.80 µmol/L (NR, 0.0-5.0 µmol/L); total protein (TP), 54.4 g/L (NR, 60-80 g/L); albumin (ALB), 26.9 g/L (NR, 35-50 g/L), indirect bilirubin and globulin were normal. White blood cell (WBC) count was 3.75 x 10^9^ /L (84.2% neutrophils, 11.5% lymphocytes, 4% monocytes, 0.3% eosinophils, 0% basophlis), and red blood cell (RBC), hemoglobin and platelet counts were within normal limits. Thrombin time was 32.2 seconds (NR, 14-21 sec); prothrombin time (PT), 22.8 sec (NR, 11-14 sec); international normalized ratio (INR) 2.35; activated partial thromboplastin time (APTT), 36.1 second (NR, 25-36 second); fibrinogen, 0.86 g/L (NR, 2-4 g/L). Erythrocyte sedimentation rate (ESR) was normal. Antinuclear antibodies and rheumatoid factor were negative. Blood culture was negative.

**Figure 1:**
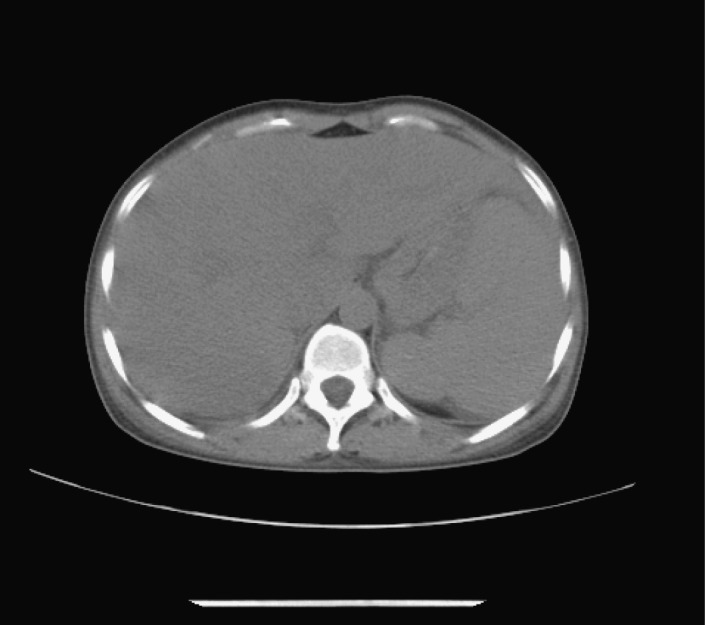
Abdominal computed tomography (CT). CT scan showed lower density of liver parenchyma and splenomegaly.

**Figure 2:**
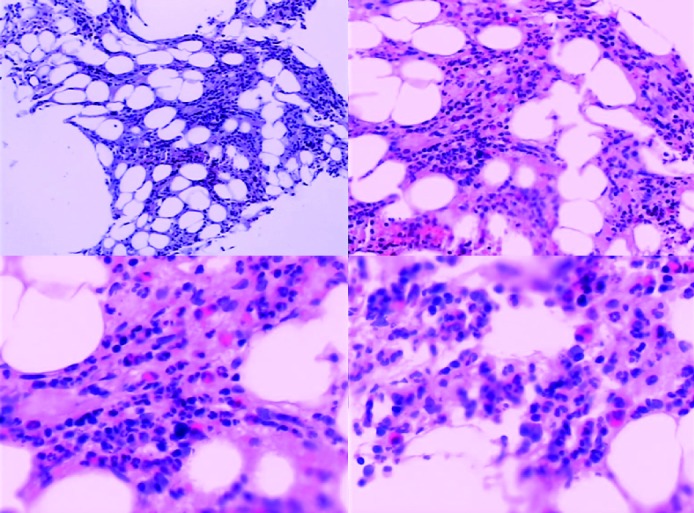
Bone marrow findings on admission. The decrease in all three hemopoietic precursors, no megakaryocytes, with mild hyperplasia of fibrous tissue with invasion of neutrophils. No apparent pathogen and malignancy were noticed.

Abdominal echography revealed homogeneous enlargement of spleen, but revealed the enlargement of the only right lobe of liver. Chest and abdominal computed tomography (CT) scan showed pachynsis of right pleura, interstitial change of lobus inferior pulmonis, and lower density of liver parenchyma and splenomegaly (Fig. 1). Serology was negative for typhus; typhoid fever; tuberculosis; syphilis; human syncytial virus; hepatitis A, B, C, D, and E virus; human immunodeficiency virus; cytomegalovirus; and Epstein-Barr virus. The patient was given antibiotics and adjuvant hepatic treatments for 1 week. The fever and maculopapules subsided, but the jaundice and liver function test still unimproved, anemia existed. Blood chemistry showed ALT 236 U/L, AST 124U/L, GGT 106 U/L, TBIL 530.6 µmol/L, DB 376.6 µmol/L. WBC count, 0.25 x 10^9^ /L (4% neutrophils, 84% lymphocytes, 12% monocytes, 0% eosinophils, 0% basophlis); RBC count, 3.37 x 10^12^ /L; hemoglobin 87 g/L; platelet 10 x 10^9^ /L. The bone marrow aspirate showed lack of red blood cell and platelet. Bone marrow biopsy revealed decrease in all three hemopoeitic precursors, no megakaryocytes were found, mild hyperplasia of fibrous tissue with invasion of neutrophil was detected, no apparent pathogen and malignancy was noticed (Fig. 2). IgM and IgG antibodies against parvovirus B19 were positive. The patient was treated with gamma globulin, liver function and bone marrow test results improved. In a follow-up visit 3 weeks after, patient was asymptomatic, the clinical blood test was normal, and liver function was normalized.

## Discussion

In this report, we describe a 34-year-old female patient, without obvious underlying disease, developed acute liver failure and myelosuppression following B19 virus infection. Parvovirus has been associated with acute hepatic failure, especially in children [9, 10], patients with parvovirus associated liver failure seems to do better than those secondary to other etiology [11]. However, cases of liver failure in adults due to parvovirus infection are rarely reported. Our case, as well as others reported in adults [12, 13], suggests that liver involvement in parvovirus B19 infection appears less severe than that in pediatric patients.

The pathophysiology of hepatic lesion in B19 infection is still unclear. Some have proposed an immunologically mediated mechanism and others have postulated a direct viral cytopathic effect on hepatocytes [1]. Basic research has demonstrated that B19 virus is capable of infecting and inducing apoptosis in both primary hepatocytes and a liver-derived cell line [14]. B19 virus induced apoptosis may proceed through a caspase 3-dependent pathway, but not the caspase 8 activity. The effect of B19 virus on HepG2 cells is a result of an apoptotic pathway initiated within the infected cell rather than as a result of an exogenous signal of a tumor necrosis factor (TNF) receptor family member [14]. A candidate molecule for affecting apoptosis in HepG2 cells is the B19 virus nonstructural protein NS1, which is a multifunctional protein with endonuclease, helicase, nucleotide triphosphate binding, and transactivating activities. B19 virus causes apoptosis of hepatocytes through NS1 expression. NS1 induces apoptosis in HepG2 cells independently of the presence of full-length viral genomic DNA or other viral proteins, this confirms that NS1 is sufficient to induce apoptosis [15]. NS1 transfection induces activation of caspases 3 and 9, these caspases are necessary for optimal induction of apoptosis. Involvement of the caspase 9 pathway is typical of apoptosis induced by internal stress, in contrast to apoptosis induced by TNF-α [15].

The anemia and myelosuppression could be secondary to parvovirus B19 infection acquired during the course of the disease. This is possible due to the marked trophism of parvovirus to the erythroid precursor [1, 16]. The most plausible explanation for the pathogenesis of myelosuppression is that some immune-mediated mechanisms induce a disturbance in the bone marrow and lead to decreased hematopoiesis. The B19 virus is well demonstrated to infect the erythroid progenitor cells and is associated with aplastic crisis in patients with chronic hemolysis [17]. The in vitro and in vivo analysis showed that the B19 virus infects mainly the erythroid progenitor cells, while other progenitor cells may also be affected by inefficient viral replication [18]. The NS1 both induces production of TNF-α and sensitizes cells to killing by TNF-α in erythroid cells [15].

Acute liver failure with myelosuppression due to parvovirus B19 infection is extremely rare in adult patients, although there are isolated cases reported on the presentation with severe hemophagocytosis and acute hepatitis associated with parvovirus infection in children [10]. We believe that parvovirus B19 infection should be considered a possible cause of liver injury, the antibody of B19 virus should be routinely tested in patients with liver dysfunction of unclear etiology.
